# Pulmonary T2* quantification of fetuses with congenital diaphragmatic hernia: a retrospective, case-controlled, MRI pilot study

**DOI:** 10.1038/s41390-025-04091-0

**Published:** 2025-09-01

**Authors:** Carla L. Avena-Zampieri, Alena Uus, Alexia Egloff, Joseph Davidson, Jana Hutter, Caroline L. Knight, Megan Hall, Maria Deprez, Kelly Payette, Mary Rutherford, Anne Greenough, Lisa Story

**Affiliations:** 1 Department of Women and Children’s Health, King’s College London, London, United Kingdom; 2 Biomedical Engineering Department, School of Biomedical Engineering and Imaging Sciences, King’s College London, London, United Kingdom; 3 Fetal Medicine Unit, Guy’s and St Thomas’ NHS Foundation Trust, London, United Kingdom; 4 University College London/Great Ormond Street Institute of Child Health, London, United Kingdom; 5 Department of Paediatric Surgery, Evelina London Children’s Hospital, London, United Kingdom; 6 Research Department of Early Life Imaging, School of Biomedical Engineering and Imaging Sciences, King’s College London, London, United Kingdom; 7 Smart Imaging Lab, Radiological Institute, University Hospital Erlangen, Erlangen, Germany

## Abstract

**Background:**

Advanced MRI techniques, motion-correction and T2*-relaxometry, may provide information regarding functional properties of pulmonary tissue. We assessed whether lung volumes and pulmonary T2* values in fetuses with congenital diaphragmatic hernia (CDH) were lower than controls and differed between survivors and non-survivors.

**Methods:**

Women with uncomplicated pregnancies (controls) and those with a CDH had a fetal MRI on a 1.5 T imaging system encompassing T2 single shot fast spin echo sequences and gradient echo single shot echo planar sequences providing T2* data. Motion-correction was performed using slice-to-volume reconstruction, T2* maps were generated using in-house pipelines. Lungs were segmented separately using a pre-trained 3D-deep-learning pipeline.

**Results:**

Datasets from 33 controls and 12 CDH fetuses were analysed. The mean ± SD gestation at scan was 28.3 ± 4.3 for controls and 27.6 ± 4.9 weeks for CDH cases.

CDH lung volumes were lower than controls in both non-survivors and survivors for both lungs combined (5.76 ± 3.59 [cc], mean difference = 15.97, 95% CI: −24.51–−12.9, *p *< 0.001 and 5.73 ± 2.96 [cc], mean difference = 16, 95% CI: 1.91–11.53, *p *= 0.008) and for the ipsilateral lung (1.93 ± 2.09 [cc], mean difference = 19.8, 95% CI: −28.48–−16.45, *p *< 0.001 1.58 ± 1.18 [cc], mean difference=20.15, 95% CI: 5.96–15.97, *p *< 0.001). Mean pulmonary T2* values were lower in non-survivors in both lungs, the ipsilateral and contralateral lungs compared with the control group (81.83 ± 26.21 ms, mean difference = 31.13, 95% CI: −58.14–−10.32, *p *= 0.006; 81.05 ± 26.84 ms, mean difference = 31.91, 95% CI: −59.02–−10.82, *p *= 0.006; 82.62 ± 36.31 ms, mean difference = 30.34, 95% CI: −58.84–−8.25, *p *= 0.011) but no difference was observed between controls and CDH cases that survived.

**Conclusions:**

Mean pulmonary T2* values were lower in CDH fetuses compared to controls and CDH cases who died compared to survivors. Mean pulmonary T2* values may have a prognostic function in CDH fetuses.

**Impact:**

- This study provides original motion-corrected assessment of the morphologic and functional properties of the ipsilateral and contralateral fetal lungs in the context of CDH.
- Mean pulmonary T2* values were lower in CDH fetuses compared to controls and in cases who died compared to survivors.
- Mean pulmonary T2* values may have a role in prognostication.
- Reduction in pulmonary T2* values in CDH fetuses suggests altered pulmonary development, contributing new insights into antenatal assessment.

## Background

Congenital diaphragmatic hernia (CDH) is a condition characterised by a defect in the diaphragm with subsequent herniation of abdominal contents into the thoracic cavity.^1^ This malformation occurs in 2.4–4.1 per 10 000 live births, with 200–300 new cases in the UK every year.^2–4^ CDH is associated with significant morbidity and mortality and over a quarter of women whose fetuses are diagnosed with the condition before 24 weeks of gestation, elect to terminate the pregnancy.^5^ For pregnancies that continue, prognosis is dependent on severity and comorbidities with survival rates ranging from 90% in those with mild disease to less than 15% in severe cases.^6^ The most common causes of mortality and morbidity are pulmonary hypoplasia and pulmonary hypertension.^7,8^ Survivors can develop chronic lung disease, have increased susceptibility to infections and gastroesophageal reflux.^6,9–12^ Impaired pulmonary function and exercise intolerance are frequently found in affected children and young adults.^13,14^

Currently, the mainstay of prenatal assessment of CDH is via ultrasound. The position of the liver and 2D assessment of the size of the contralateral lung is evaluated, often as a ratio with head biometry as a tool to predict adverse outcome.^15^ Several measurements have been evaluated, including the lung to head ratio,^16^ the observed to expected lung to head ratio (o/e LHR),^17^ the quantitative lung index^18^ and the lung-to-liver signal intensity ratio,^19^ to determine their prognostic value for postnatal morbidity.^20–24^ All those assessments, however, only focus on the contralateral lung, as the ipsilateral is often poorly visualised on 2D ultrasound.^25^ Although 2D assessment has been found to correlate well with 3D volume for the contralateral lung,^26,27^ the results are all only indirect assessments of the size of the lungs.

MRI is increasingly used in clinical practice to assess CDH severity as an adjunct to ultrasound, due to its excellent soft tissue resolution and contrast^28^; it can provide enhanced evaluation of the location of the defect, position of herniated organs and their impact on surrounding structures and provides a consistent assessment of lung volumes on both the ipsilateral and contralateral sides.^29–31^ MRI has been shown to provide accurate measurements of total lung volume and the ratio of the volume of the liver herniated into the thoracic cavity to the volume of the thoracic cavity.^32–34^ MRI measurements of the observed to expected total lung volume were found to be a more accurate predictor of postnatal survival than the observed to expected lung-to-head ratio.^30,35^ However, there are no MRI-based techniques in current clinical use that provide information on pulmonary tissue properties likely to determine subsequent function.

The advent of advanced fetal MRI techniques such as motion-correction and T2* relaxometry, now facilitates indirect assessment of tissue perfusion/oxygenation in addition to anatomical evaluation. T2* relaxation relates to the decay of transversal magnetisation due to the inherent inhomogeneity of the magnetic field. It thereby exploits the blood oxygen level–dependent (BOLD) effect and utilises the fact that oxygenated and deoxygenated haemoglobin have different paramagnetic properties, providing an indirect assessment of perfusion/oxygenation. Mean T2* values can be quantified for specific regions of tissue, and T2* maps generated, highlighting regional variations in oxygenation, with areas of lower oxygen concentration appearing as regions with lower signal intensity in the T2* maps. Although T2* relaxometry has been applied to the fetal lungs in a low-risk population^36–39^ and in fetuses at risk of preterm birth,^40^ to our knowledge no studies to date have utilised the technique for evaluation of the lungs in fetuses with CDH. This study aimed to assess whether lung volumes and mean pulmonary T2* values were lower in fetuses with CDH in comparison with a control cohort of uncomplicated pregnancies and to evaluate if differences occurred between fetuses who survived, and those who did not.

## Methods

A retrospective case-controlled study was performed over a six-year period from 2017 to 2023. Datasets were selected if women underwent a fetal MRI due to a diagnosis of CDH and had given consent for their data to be used for research (“Quantification of fetal brain growth and development using MRI” [REC 07/H0707/105]). A control group was selected from pre-existing datasets where women had been imaged as part of two ethically approved research studies as control participants: “Placenta Imaging Project” [REC: 16/LO/1573] and “Cardiac-Placenta imaging in pregnancy (CARP)” [REC: 08/LO/1958]) and had no adverse neonatal outcomes.

Exclusion criteria for both groups included: pregnancy complications such as gestational diabetes or pre-eclampsia, chromosomal abnormalities in any of the cases, or incomplete outcome data for the control group. The exclusions in the CDH cohort, over the period analysed, were due to a lack of consent rather than incomplete data as only two additional clinical MRI scans were performed for CDH during the study period, but these women did not consent for their data to be used for research.

All participants had undergone a fetal MRI scan on an Ingenia 1.5T (Philips) imaging system. Maternal heart rate and intermittent blood pressure readings were performed during the scan. T2-weighted single-shot turbo spin echo (ssTSE) sequences were acquired in multiple orthogonal planes. Repetition time (TR) = 15,000 ms, Time to Echo (TE) = 80/100 ms, voxel size 1.25 × 1.25 mm, slice thickness 2.5 mm and spacing 1.25 mm. A multi-echo gradient echo single-shot echo planar sequence was then performed to obtain T2* images with the following parameters: 3 mm^3^ resolution, five echo times [11.4 ms/57.8 ms/104.2 ms/150.6 ms/197.0 ms], TR = 3 s, parallel imaging SENSE factor = 3, flip angle = 90°. The field of view was set to 360 mm × (320–400) mm × (60–120) mm using a coronal imaging plane aligned to the scanner co-ordinates, in order to encompass the whole thorax and minimise artefacts from parallel imaging reconstruction techniques.

Using an in-house pipeline, deformable slice to volume reconstruction (DSVR)^36^ was first used to yield 3D motion-corrected high resolution anatomical fetal body T2 weighted (T2w) datasets as previously validated.^41,42^ This post-processing method of motion correction is useful in rectifying both in and out-of-plane local distortions within the fetal organs allowing enhanced accuracy and consistency of lung segmentations in 3D space. The resulting 3D DSVR reconstructed images of the fetal body had 0.75 mm (T2w) isotropic resolution and were globally reoriented to the standard radiological space.

**Fig. 1:**
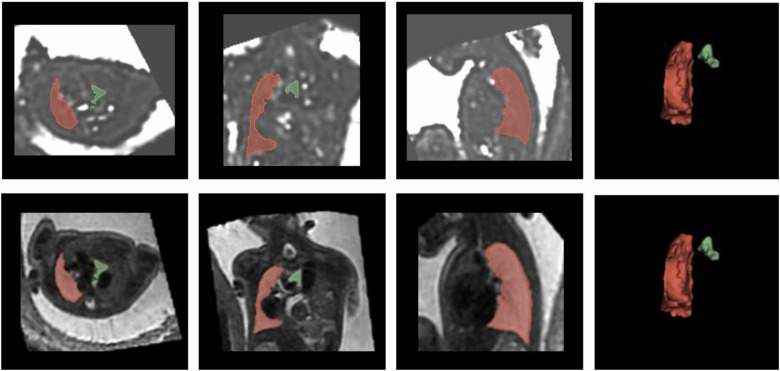
Manual lung segmentation of a 35 + 3-week fetus with a left-sided congenital diaphragmatic hernia, in axial, coronal and sagittal plus 3D segmentation on a T2* (top) and T2 (bottom) image after applying deformable slice to volume reconstruction. The left lung is represented in green and the right lung in red.

An in-house Python script was used to generate T2* maps using mono-exponential decay fitting.^43^ We also applied DSVR to T2* datasets as previously described^37,44^ with resulting images of 1.2 mm (T2*) isotropic resolution. The T2* images were registered to T2w images, with manual input when needed, using a combination of rigid and non-rigid registration in MIRTK toolbox^45^ which allowed for alignment of T2* images with T2w images. 3D images with satisfactory motion-correction and quality, with complete data for both lungs, were selected. Good intra and inter observer variability have previously been confirmed.^38^ Good agreement between automated and manual fetal lung segmentation has also been confirmed previously with a high Dice score of 0.9.^46,47^ The fetal lungs were segmented separately manually for the CDH cohort and using a pre-trained 3D deep learning pipeline^48^ for the control cohort, followed by manual refinement by an experienced operator (CAZ) avoiding any surrounding structures such as the pulmonary hilum and the thymus as well as non-pulmonary tissue such as maternal structures, and amniotic fluid (Fig. 1).

Mean T2* values and lung volumes were obtained using a purpose-built Python script.^43,49^ Cases were excluded where datasets were found to be corrupted or where excessive motion was present. Details of maternal demographic data, antenatal ultrasound scans, whether fetal endotracheal occlusion (FETO) was performed, and pregnancy complications were recorded from the medical records with participant consent. All fetuses in this study were imaged at St Thomas Hospital, however, they delivered in three different tertiary units. All of the neonatal units had similar protocols in neonatal management. One of the CDH cases included was in a DCDA twin pregnancy (the other twin was unaffected). Pregnancy outcome, delivery parameters and maternal and neonatal outcome data were collected until discharge from hospital from the medical records. Neonatal outcome parameters assessed included: gestational age at delivery, sex of infant, birthweight, neonatal unit admission, numbers of days of invasive ventilation, need for continuous positive airway pressure, supplementary oxygen support (high or low flow), development of respiratory distress syndrome (RDS), persistent pulmonary hypertension of the newborn (PPHN), pulmonary hypertension, use of Extra Corporeal Membrane Oxygenation (ECMO) and details of postnatal surgery.

### Statistical analysis

Data were first checked for normality. Demographic and outcome parameters between groups were assessed for statistical significance using the Student’s *t* test where data were continuous and Chi-squared where categorical. Mean pulmonary T2* values and lung volumes were compared between the control group and CDH cohort using linear regression to account for the effects of gestational age. All statistical analysis was undertaken using SPSS version 29.0.1 (SPSS IBM).

## Results

Twelve datasets from the CDH cohort and 34 from the control cohort initially met the criteria for inclusion into the study. One control was subsequently excluded due to significant motion corruption which could not be compensated for with post processing pipelines. Twelve CDH datasets and 33 controls were therefore suitable for final analysis. Details of clinical characteristics of the two groups are summarised in Table 1. Nine of the CDH cases had left and three right sided hernias, none were bilateral.

**Table 1:** Clinical characteristics of the control and congenital diaphragmatic cohorts.

	Control	Congenital diaphragmatic hernia	*p*-value
	*n* = 33	*n* = 12	
Maternal age (years)			0.02
Mean (SD)	34.4 (4.0)	30.6 (6.2)	
Range	26–44	21–42	
BMI (kg/m^2^)			0.628
Mean (SD)	27.6 (6.2)	28.6 (5.7)	
Range	16.8–43.3	20.7–37	
Ethnicity *n* (%)			0.218
White	26 (79%)	7 (78%)	
Mixed	0 (0%)	1 (11%)	
Asian	2 (6%)	0 (0%)	
Black	4 (12%)	0 (0%)	
Other	1 (3%)	1 (11%)	
Gestational age at scan (weeks)			0.645
Mean (SD)	28.3 (4.3)	27.6 (4.9)	
Range	21.6–35.7	21.6–35.5	
Gestational age at delivery (weeks)			<0.001
Mean (SD)	38.9 (7.3)	37.9 (1.4)	
Range	37–42.1	34.6–39.5	
Sex of fetus *n* (%)			0.453
Male	12 (38%)	7 (54%)	
Female	20 (63%)	6 (46%)	
Birthweight (g)			<0.001
Mean (SD)	3373 (736)	2792 (669)	
Range	2780–4440	1420–3870	
Birthweight centiles *n* (%)			0.346
0–3	0 (0%)	2 (17%)	
03–10	0 (0%)	2 (17%)	
10–25	1 (3%)	3 (25%)	
25–50	12 (38%)	3 (25%)	
50–75	9 (28%)	0 (0%)	
75–90	5 (16%)	0 (0%)	
90–97	4 (12%)	2 (17%)	
97–100	1 (3%)	0 (0%)	
Parity *n* (%)			0.004
0	18 (55%)	1 (8%)	
1	11 (33%)	3 (25%)	
2	2 (6%)	5 (42%)	
3	1 (3%)	2 (17%)	
4	0 (0%)	1 (8%)	
5	0 (0%)	0 (0%)	
6	0 (0%)	0 (0%)	
7	1 (3%)	0 (0%)	

**Fig. 2:**
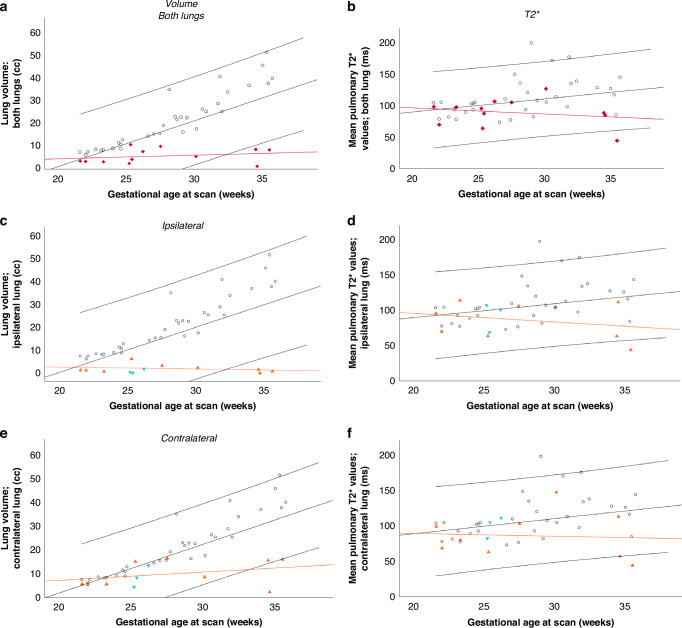
Lung volume and mean pulmonary T2* values related to gestational age at scan in controls (○ controls) and right-sided CDH cases () and left-sided CDH cases (). **a** Lung volume in both lungs combined (CDH cohort). **b** T2* values in both lungs combined. **c** Volume in the ipsilateral lungs. **d** T2* values in the ipsilateral lung. **e** Volume in the contralateral lungs. **f** T2* values in the contralateral lungs. Associated subgroups best fit lines are displayed with black representing the control group and their 95% confidence intervals and orange the left-sided CDH group.

Lung volume of both lungs combined, the ipsilateral, and contralateral lungs were significantly lower in the CDH cohort than in the control group (Table 2, Fig. 2a, c, e). Mean pulmonary T2* values were also lower in the CDH cases than in controls for both lungs combined, the ipsilateral and the contralateral lung (Table 2, Fig. 2b, d, f).

**Table 2:** Table of results of both lungs, the ipsilateral and the contralateral volumes and mean pulmonary T2* values of the congenital diaphragmatic hernia cohort (all cases, left-sided only and survivors and non-survivors) in comparison to the controls.

CDH (all)	Mean +/− SD	Mean difference	95% CI	R^2^	*p*-value
Lung volume (cc)					
Both lungs combined	5.7 +/− 3.19	15.98	9.91–19.39	0.73	<0.001
Ipsilateral lung	1.78 +/− 1.71	19.95	13.76–23.55	0.75	<0.001
Contralateral lung	9.71 +/− 5.25	12.02	5.93–15.34	0.70	<0.001
Pulmonary T2* (ms)					
Both lungs combined	88.97 +/− 21.76	23.99	3.91–41.43	0.20	0.019
Ipsilateral lung	88.02 +/− 24.00	24.94	4.50–42.87	0.20	0.017
Contralateral lung	89.92 +/− 29.18	23.04	1.79–41.51	0.19	0.033
Left sided CDH	Mean +/− SD	Mean difference	95% CI	R²	*p*-value
Lung volume (cc)					
Left lung	2.07 +/− 1.86	17.01	11.61–22.29	0.70	<0.001
Right lung	10.04 +/− 5.68	14.35	8.43–20.12	0.70	<0.001
Pulmonary T2* (ms)					
Left lung	86.36 +/− 25.92	24.56	2.27–46.73	0.16	0.032
Right lung	86.58 +/− 32.62	28.42	5.39–51.29	0.23	0.017
Survivors/non-survivors	Mean +/− SD	Mean difference	95% CI	R²	*p*-value
Lung volume (cc)					
Non-survivors					
Both lungs combined	5.76 +/− 3.59	15.97	−24.51–−12.90	0.74	<0.001
Ipsilateral lung	1.93 +/− 2.09	19.80	−28.48–−16.45	0.75	<0.001
Contralateral lung	9.6 +/− 5.87	12.13	−20.66–−9.23	0.73	<0.001
Survivors					
Both lungs combined	5.73 +/− 2.96	16.00	−58.14–−10.32	0.87	0.008
Ipsilateral lung	1.58 +/− 1.18	20.15	−59.02–−10.82	0.88	<0.001
Contralateral lung	9.90 +/− 4.90	11.83	−58.84–−8.25	0.87	0.294
Pulmonary T2* (ms)					
Non-survivors					
Both lungs combined	81.83 +/− 26.21	31.13	−58.14–−10.32	0.25	0.006
Ipsilateral lung	81.05 +/− 26.84	31.91	−59.02–−10.82	0.25	0.006
Contralateral lung	82.62 +/− 36.31	30.34	−58.84–−8.25	0.23	0.011
Survivors					
Both lungs combined	98.96 +/− 7.63	14	−23.44–29.71	0.22	0.813
Ipsilateral lung	97.78 +/− 17.32	15.18	−22.72–31.72	0.21	0.739
Contralateral lung	100.14 +/− 11.87	12.82	−24.88–28.39	0.22	0.895

In the control cohort, lung volumes positively correlated with gestational age on both left and right lungs (left: 95% CI: 2.08–2.9, *p *< 0.001; right: 95% CI: 2.49–3.38, *p *< 0.001). Mean pulmonary T2* values also increased with gestational age in the control cohort in both left and right lungs (left: 95% CI: 0.33–5.15, *p *= 0.027; right: 95% CI: 1.27–5.96, *p *= 0.004).

For left sided CDH cases, lung volumes were lower than controls for both the left and right lungs. Mean pulmonary T2* values were lower than the CDH cases than in the control cohort in the left and right lungs.

In the right-sided CDH group, numbers were too small for analysis. Results can be seen in Fig. 2.

### Outcomes

Fifty eight percent (*n* = 7) of the CDH cohort, died in the neonatal period. Four died in the first 48 hours because of severe pulmonary hypoplasia. Six cases had postnatal surgical repair of the hernia, two right-sided and four left-sided. One died post-operatively. One case had FETO at 26 weeks of gestation. The MRI for this case was obtained pre-FETO and the neonate subsequently died due to complex congenital heart disease (CHD) including left atrial isomerism. The majority of the CDH infants had pulmonary hypertension (66%; Table 3) and two of seven survivors had persistent pulmonary hypertension of the newborn (PPHN). According to classification of severity established by the o/e LHR where low values were associated with extreme pulmonary hypoplasia and low survival rates^50^ (15–25%: severe pulmonary hypoplasia; 26–45%: moderate pulmonary hypoplasia; >45%: mild pulmonary hypoplasia), six cases had severe pulmonary hypoplasia, one moderate and three had mild pulmonary hypoplasia, two ratios were not recorded as the data were from other hospitals (Supplementary Table S1).

**Table 3:** Length of stay, respiratory support and outcomes of CDH infants.

	Non-survivors (*n* = 7)	Survivors (*n* = 5)	*p*-value
Length of stay neonatal unit (Median, Q1–Q3)	2 (1.5–7.5)	40 (26–42)	0.003
Length of respiratory support (Median, Q1–Q3)	2 (1–9)	22 (21–28)	0.028
Length of oxygen supplementation (high-flow or O_2)_ (Median, Q1–Q3)	0 (0–1.5)	6 (5–11)	0.118
Respiratory distress syndrome	2	1	
Pulmonary hypertension	5	3	
Persistent pulmonary hypertension of the newborn	2	0	
Herniation of the liver in chest	5	1	
Pulmonary sequestration	1	0	
Cardiac abnormalities	1 complex congenital heart disease, 2 coarctation of the aorta	0	
Pleural effusion	0	1	
Extracorporeal membrane oxygenation	0	0	

Echo data were available for nine cases, another case died in the delivery room before this examination could be performed. From survivors with echo data (*n* = 4) only one baby did not have any type of ventricular dysfunction, two had impaired biventricular function and one had impaired left ventricular function. In non-survivors with echo data (*n *= 5), all but one with normal biventricular function, had some type of ventricular dysfunction, one left and two right impaired ventricular function and one had ventricular discordance.

**Fig. 3:**
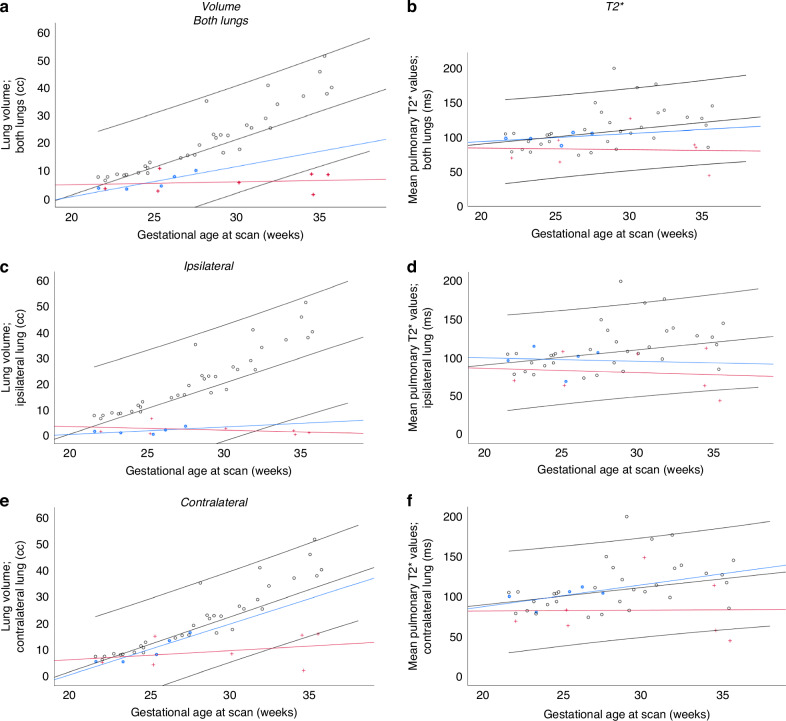
Lung volume and mean pulmonary T2* values related to gestational age at scan in controls (○controls), fetuses who survived () and fetuses who did not survive (). **a** Volume in both lungs combined. **b** T2* values in both lungs combined. **c** Volume in the ipsilateral lungs. **d** T2* values in the ipsilateral lungs. **e** Volume in the contralateral lungs. **f** T2* values in the contralateral lungs. Associated subgroups best fit lines are displayed with black lines representing the control group and their 95% confidence intervals, blue the fetuses who survived and red those who did not.

The volume of both lungs combined and the ipsilateral lung (Table 2, Fig. 3a) were lower in the CDH cases than controls irrespective of whether they survived or not. However, the contralateral lung volume (Table 2, Fig. 3e) was only smaller in comparison with the control group in CDH cases that did not survive.

Mean pulmonary T2* values of both lungs combined, ipsilateral and contralateral lungs (Table 2, Fig. 3b, d, f) were lower in CDH cases that did not survive in comparison to controls. No significant difference was observed in survivors in both lungs combined, the ipsilateral and the contralateral lung.

## Discussion

We have demonstrated that both pulmonary volumes and mean pulmonary T2* values were significantly lower in CDH fetuses compared to the controls. The finding of lower lung volumes in both lungs is in accordance with published literature.^51–57^ Our segmentations were performed on motion-corrected aligned T2*-T2 datasets to avoid including vasculature, so were likely to be more conservative than previous studies, which used non-motion corrected T2-derived volumes.^51–57^ Post-processing techniques utilised here are likely to have resulted in more reliable volumetric and T2* values as motion-correction pipelines result in increased accuracy of volumes and conservative segmentations will reduce partial volume effects. Motion correction has only been used by one other study.^57^ However overall, the lung volumes generated in this study were comparable to those published in the literature.^51–57^

The contralateral lung is commonly used for assessment of pulmonary volume, particularly when ultrasound is used where the ipsilateral lung is often not visualised.^25,58,59^ Several studies have evaluated both lungs using ultrasound and MRI.^51,53,60,61^ Two volumetric MRI studies evaluated the prediction of outcomes based on the ipsilateral lung volume and found lower prognostic accuracy for survival, the need for ECMO and development of chronic lung disease in comparison with the contralateral lung volume.^51,62^ In contrast, one 3D ultrasound study demonstrated that total lung volume was a more accurate predictor of perinatal mortality than the ipsilateral or contralateral lung individually.^60^ In our study, the lung volumes of both lungs combined and of the ipsilateral lung were lower than controls in both survivors and non-survivors. In infants who survived, the contralateral lung volume was not significantly different from the control cases. The ipsilateral lungs, being directly compressed by the herniated viscera, are typically more hypoplastic than the contralateral lungs^63^ often resulting in compromised pulmonary vasculature.^64,65^ The degree to which the contralateral lung is compressed is related to the size of the defect and in this series the cases had different degrees of severity.

Mean pulmonary T2* values were significantly lower in both the ipsilateral and contralateral lungs in comparison with the control cohort. Previous studies have explored normal pulmonary development at different field strengths using this technique.^37–39,66^ The control fetuses in our study, imaged between 21 and 36 weeks of gestation demonstrated an increase in mean pulmonary T2* values with advancing gestation. This aligns with our previous findings that pulmonary mean T2* values increase with advancing gestational age.^38^ Although, other studies with narrower gestational age ranges, fewer cases, and different field strengths have reported conflicting trends with two reporting an increase over gestation, two showed no trend and one a decrease over gestation.^37–39,66^ This increase in mean pulmonary T2* values observed in the present study may be attributable to an increase in metabolic demand and increased angiogenesis as gestation increases, both of which are known to alter T2* values.^67^

No studies have previously evaluated mean pulmonary T2* values in fetuses with CDH however, lung signal intensities and T2 values have previously been quantified.^68^ Cannie et al found that T2 values were significantly lower in a group of 11 left-sided CDH cases compared to 105 controls and that the ipsilateral values were lower than the contralateral results.^68^ Similar findings were observed in an ovine model of surgically induced left-sided CDH. Pulmonary T2 values were significantly lower in both lungs, however, this was most marked in the left lung.^69^ T2 values correlated with lung weight and the presence of pulmonary hypoplasia at autopsy,^69^ with the ipsilateral lung again being more affected than the contralateral lung. During fetal lung development, T2 values of healthy fetuses are known to increase with gestation, reflecting maturation of lung tissue with increased surfactant production and increased complexity of the alveolar structure.^68,70^ T2* values are the result of the combined effects of spin-spin relaxation (T2) and magnetic field inhomogeneity^67^ and reflect tissue properties such as tissue density and structural integrity which can indicate structural changes across gestation. T2 and T2* values are therefore intrinsically linked, both representing changes in tissue morphology across gestation. The finding of a reduction in pulmonary T2* values in CDH fetuses in our study and lower pulmonary T2 values in both animal models and fetal studies indicates altered pulmonary development. Blunted microvascular growth has been reported in CDH rat,^71^ mice^72^ and rabbit^73^ models.^74^ Pulmonary arteries exhibit hypertrophy, with an increased number of contractile vascular smooth muscle cells, extending more distally than in control cases with a reduction in alveolar number and overall vascular branching.^73,75–78^ Alterations in pulmonary vasculature will also affect pulmonary T2* values and could lead to impaired pulmonary blood flow, which may contribute to the observed reductions in mean T2* values, particularly in areas with poorer perfusion. Alterations in metabolism may also account for the lower mean pulmonary T2* values observed in the CDH cases in this study. A rat model of CDH reported reduced adenosine triphosphate (ATP) and increased lactate levels, indicating a transition to anaerobic metabolism in the hypoplastic lungs in the CDH pups compared with controls.

### Strengths and limitations

To our knowledge this is the first study to evaluate pulmonary development using T2* relaxometry and motion correction pipelines in cases of CDH providing an indirect assessment of the functional properties of pulmonary tissue. The datasets for the CDH fetuses were analysed retrospectively so the exclusions were due to a lack of consent during that time-period rather than incomplete data, making the sample size one of feasibility. Due to the small numbers, it was not possible to evaluate the MRI findings with the clinical severity of CDH as diagnosed on ultrasound or to correlate findings robustly with neonatal and longer-term outcomes. Due to the retrospective nature of the study, o/e LHR was the only measure available for classifying CDH severity based on ultrasound data but further exploration of additional factors could be a focus for future research. Numbers were also too small to determine whether T2* and volume combined enhanced analysis, but this is something that could be explored in future studies.

Whilst fetal ultrasound remains the mainstay of assessment of the fetal lungs in cases of CDH in clinical practice, MRI is increasingly being utilised due to the fact it can provide visualisation and volumes of both the contralateral and ipsilateral lungs, the latter being more difficult to assess on ultrasound. In addition, it can also identify the exact position of abdominal organs such as the liver which has been shown to enhance prognostication.^79^ In the research setting, MRI has the potential to provide even more insight into pulmonary development. T2* relaxometry provides an indirect assessment of tissue perfusion/oxygenation, thereby providing insight into more functional properties of tissue which can be used in cases of CDH to explore alterations in lung development further. This study has revealed that low T2* values in the ipsilateral lung may have the potential to predict fetuses that may not survive postnatally. Although MRI scans do have increased cost in comparison with ultrasound advances in low field scanning, which is particularly well suited to T2* relaxometry,^80^ mean that accessibility may increase in the future making this a potentially useful clinical tool.

In this pilot series three cases had congenital heart disease (CHD), which could further impact pulmonary development and consequently T2* values. The impact of the CHD on the CDH T2* values is therefore not clear. However, the two conditions are often concomitant and whether they exist together or independently, clinicians need to be able to have tools available to counsel parents on likely respiratory outcomes postnatally. Further work in adequately powered studies would be needed to assess subgroups. We were, however, able to demonstrate significant differences in T2* values between those cases and the controls as well between those who survived and those who died. Future studies could benefit from longitudinal measurements across multiple time points during pregnancy to better capture the trajectory of mean T2* values, particularly for prognostication purposes. These findings offer a preliminary exploration that highlights areas for further investigation in larger cohorts. Future prospective studies are therefore essential to expand on these findings and to explore their clinical implications.

## Conclusion

Overall, lower mean pulmonary T2* values were observed in the lungs of fetuses with CDH compared to controls. In the CDH fetuses, lung volumes were lower than controls for both lungs and the ipsilateral lung in both survivors and non-survivors. However, mean pulmonary T2* values were only lower in non-survivors, with survivors showing no significant difference from controls. These are preliminary findings, and a larger sample size will be required to explore these observations in more detail. Future studies could also benefit from exploring the relationship between T2* values and fetal ultrasound measurements, such as the O/E LHR and MRI-derived O/E TFLV, to further validate T2* in the evaluation of CDH. However, the results provide promising insights into the potential for further investigation of pulmonary alterations in CDH using advanced imaging techniques.

## Supplementary information

Supplementary Table S1

## Data Availability

The datasets generated during and/or analysed during the current study are available from the corresponding author on reasonable request.
