# Supplementary material for: Pulmonary T2* quantification of fetuses with congenital diaphragmatic hernia: a retrospective, case-controlled, MRI pilot study
Source: Pediatr Res. 2025 Sep 1;99(6):2287–95. doi: 10.1038/s41390-025-04091-0 (PMC13433260; doi:10.1038/s41390-025-04091-0)
Supplement: Supplementary file 1 [file 41390_2025_4091_MOESM1_ESM.pdf]

Supplementary Table S1:

	O/E USS (%)	O/E TFLV at MRI (%)
1	Not recorded	8%
2	Pre FETO 18%<br>Post FETO 21%	14%
3	34%	23%
4	46%	9%
5	21%	25%
6	48%	10%
7	Not recorded	14%
8	<25%	10%
9	75%	20%
10	<25%	1%
11	25%	36%
12	24%	18%

Supplementary S1: Score of each case of the observed to expected lung to head ratio at ultrasound and observed to expected total fetal lung volumes at MRI .
